# Periaqueductal Grey differential modulation of Nucleus Accumbens and Basolateral Amygdala plasticity under controllable and uncontrollable stress

**DOI:** 10.1038/s41598-017-00562-5

**Published:** 2017-03-28

**Authors:** Omer Horovitz, Alon Richter-Levin, Lin Xu, Liang Jing, Gal Richter-Levin

**Affiliations:** 1 Department of Psychology, University of Haifa, Haifa, Israel; 2 The Institute for the Study of Affective Neuroscience (ISAN), Haifa, Israel; 3 Sagol Department of Neurobiology, University of Haifa, Haifa, Israel; 4 Key Laboratory of Animal Models and Human Disease Mechanisms, and KIZ/CUHK Joint Laboratory of Bioresources and Molecular Research in Common Disease, and Laboratory of Learning and Memory, Kunming Institute of Zoology, the Chinese Academy of Sciences, Kunming, 650223 China; 5 University of the Chinese Academy of Sciences, Beijing, 100049 China; 6 CAS Center for Excellence in Brain Science, 320 Yue Yang Road, Shanghai, 200031 China

## Abstract

Resilience has been conceptualized in part as a dynamic process that includes the ability to adapt to stressful conditions. As such it encompasses the extent to which neural plasticity may be promoted. The current study examined *metaplasticity* by referring to the “plasticity of synaptic plasticity” in a neural circuit composed of the basolateral amygdala (BLA) and the nucleus accumbens (NAcc), using behavioural stress controllability with or without preceding stimulation of the dorsal periaqueductal gray (i.e. dPAG priming). A tendency for increased plasticity in the controllable versus the uncontrollable group was found in both the BLA and NAcc. dPAG priming suppressed NAcc LTP in all groups, but it suppressed BLA LTP only in the uncontrollable group, demonstrating dissociation between either controllable and uncontrollable groups or the NAcc and BLA. Thus, metaplasticity in the dPAG-BLA-NAcc circuit regulated differentially by controllable or uncontrollable stress may underlie stress coping, and thus contribute to stress-related psychopathologies.

## Introduction

Stressful experiences promote behavioural and neural changes that may be of adaptive value, but may sometimes also increase the occurrence of negative outcomes^1, 2^. Studies indicate that individuals’ perceptions of their ability to contend with a stressful experience may affect their choice of coping strategies, and have a profound effect on the likelihood of developing stress-related psychopathologies^3^. In fact, psychological factors such as predictability, avoidability, or escapability of an aversive stimulus are found to be more significant than the nature of the aversive stimulus itself in determining the outcome^4^. It was proposed that behavioural controllability under all sorts of so called stress should be formulated into the definition of stress *per se* as relating to psychopathology^5^.

Behavioural control effects in response to a stressful experience have been extensively studied in the psychological field for decades^6–8^. The ‘triadic design’ (i.e. controllable stressor, yoked-uncontrollable stressor, or no stressor), and the associated differences in levels of “learned helplessness” effects, is a prominent example of such exploration^9, 10^. In animals, studying the effects of behavioural controllability involves exposure to the ‘two-way shuttle avoidance’ (TWSA) task. Animals are allocated at random and are exposed to either controllable (escapable) or uncontrollable (inescapable) aversive stimuli (usually footshocks). Following exposure, their behaviour in response to the aversive stimuli is assessed. In this setting the controllable group is presented with a series of tone-shock cycles, and shuttling to the other compartment of the maze terminates the cycle and enables them to either escape or avoid the foot-shock. The uncontrollable group is presented with exactly the same stimuli as the controllable group, but in contrast their behaviour has no effect on the outcome, which is either induced by the performance of the controllable partner or is pre-set by the computer according to the parameters of the controllable group. Thus, the only difference between the groups is the controllability over the aversive stimuli being experienced^11^. Since the pioneering work of Seligman & Maier^10^ on maladaptive responses in the face of uncontrollable stress, an entire body of research has been dedicated to elucidate the mechanisms associated with the effects of stressors controllability or the lack of it^8^. Profound behavioural, physiological, neural, and immunological deficits as a result of exposure to uncontrollable stress have been reported, affecting activity^8, 12–14^, aggression^15, 16^, affectivity^8^, motivation^17^, and learning and memory capabilities^10, 13, 18^. Furthermore, there has been progress in identifying the neural mechanisms associated with these behavioural alterations. For example, the prefrontal cortex and neuro-modulation by serotonin from the dorsal raphe nucleus and by corticotrophin-releasing factor have been implicated with these alternations^19^.

Many of the studies of controllable/uncontrollable stress were performed implementing multiple exposures to aversive stimuli within a single day. Repeated exposure to cycles of tone-shock enables animals to acquire the avoidance task and towards the end of such a training day, animals may display clear levels of escape and avoidance responses, indicating that they have gained a level of controllability over the challenge^12^. However, if these animals are brought back to the TWSA, they still exhibit high levels of contextual freezing, indicating that while they did gain a level of operational controllability they are still afraid of the context^12^. We found that prolonged training in the TWSA may lead to the development not only of operational controllability, but also of emotional controllability, i.e., the suppression also of the contextual fear response^12^. Interestingly, the development of emotional controllability was found to be associated with the development of stress resilience, while the prolonged uncontrollable stress exposure was found to lead to a lasting state of learned helplessness, with symptoms of depression^12, 20^. In the present study we will focus on the impact of prolonged exposure to controllable or uncontrollable stress on behaviours and synaptic plasticity.

Learning and memory processes in the brain have a close association with the activity of *positive/negative* affective neural circuits^21–23^. Typically however, *positive* neural circuits such as those involving the striatum and specifically the Nucleus Accumbens (NAcc) have been mainly studied in appetitive/addictive learning conditions^24, 25^ while *negative* circuits such as those involving the amygdala have been traditionally studied separately and in relation to aversive/withdrawal learning^26, 27^. One study indicated on increased ERK2 and CREB activation in the BLA in the uncontrollable group compared with the controllable and naïve groups^12^. In line with this finding, other studies have shown that lesioning the BLA reverses the previously escape-malfunctioning of uncontrollable rats^28^. Similarly, different controllability levels modulated activity and plasticity in the hippocampus and the BLA simultaneously^14^. Briefly, the authors reported that uncontrollable stress enhances neural plasticity in the hippocampus and increases baseline responses in the amygdala^14^. Fewer studies have examined the effects of behavioural controllability on positive affect related brain structures. Among these, some studies have reported an interaction between the NAcc and levels of behavioural controllability through highlighting opposite responses of mesolimbic dopamine system to controllable and uncontrollable aversive experiences^29^. Other studies have documented a modulation of serotonin efflux but not of dopamine efflux in the NAcc shell following different stressor controllability levels^30, 31^. It is worth noting that NAcc functions are not restricted to appetitive stimuli only, as these functions have been shown to also to aversive stimuli (for a review, please see: ref. 32). Recent evidence also suggests an analogous picture with regards to the BLA function and appetitive stimuli (for example, see: ref. 33).

Modulation of activity of *positive/negative* affective neural circuits by higher limbic regions such as the prefrontal cortex (PFC) - (Appetitive learning: ref. 34; Aversive learning: ref. 35) and the hippocampus (Appetitive learning: ref. 36; Aversive learning: ref. 37) has been indicated. Behavioural controllability was shown to have a significant effect on these regions as well. For example, an immunohistochemistry study indicated that stressor controllability modulates stress-induced decreases in neurogenesis and increases in fibroblast growth factor-2 in a rats hippocampus^30, 31^. A sex dependent effect for controllability with a modulation of hippocampal neurogenesis in males but not in females was also reported^38^. A follow-up study attributed the effects of different levels of stressor controllability on the NAcc to a modulation by the dorsal raphe nucleus^39^. However, the possibility of brain stem modulation of *positive/negative* affective neural circuits has so far gained less attention.

Furthermore, so far behavioural controllability effects on brain regions associated with either positive or negative affect have been studied separately. The *simultaneous* effects of behavioural controllability on both positive and negative affect circuits have not been addressed. Recently, dorsal periaqueductal gray (dPAG) simultaneous modulation of ventral subiculum induced-plasticity in both the BLA and NAcc was reported^40, 41^. This places the dPAG as a candidate for simultaneously modulating both positive and negative affect circuits.

The current study was designed to assess the long-term behavioural manifestations of exposure to prolonged controllable or uncontrollable stress and the simultaneous electrophysiological reflections in *positive/negative* affective brain circuits. Furthermore, we have examined the modulation by dPAG priming of plasticity in *positive/negative* affective brain pathways under controllable/uncontrollable stress conditions.

## Results

### Behavioural results

#### Weights

A one-way ANOVA for averaged weights prior to the electrophysiological assessments did not reveal any significant effects between groups (i.e. Naïve, Controllable and Uncontrollable animals), [F_(2,38)_ = 0.378, *n.s*.].

### Novel setting exploration

Independent student *t*-tests for a 10 min average number of free ‘side to side’ transitions before the exposure to the TWSA training in the 1^st^ day did not reveal any significant differences between the controllable group (21.33 ± 0.95; *n* = 18) and the uncontrollable group (22.07 ± 1.16; *n* = 13).

### ‘TWSA’ task performances

**Figure 1:**
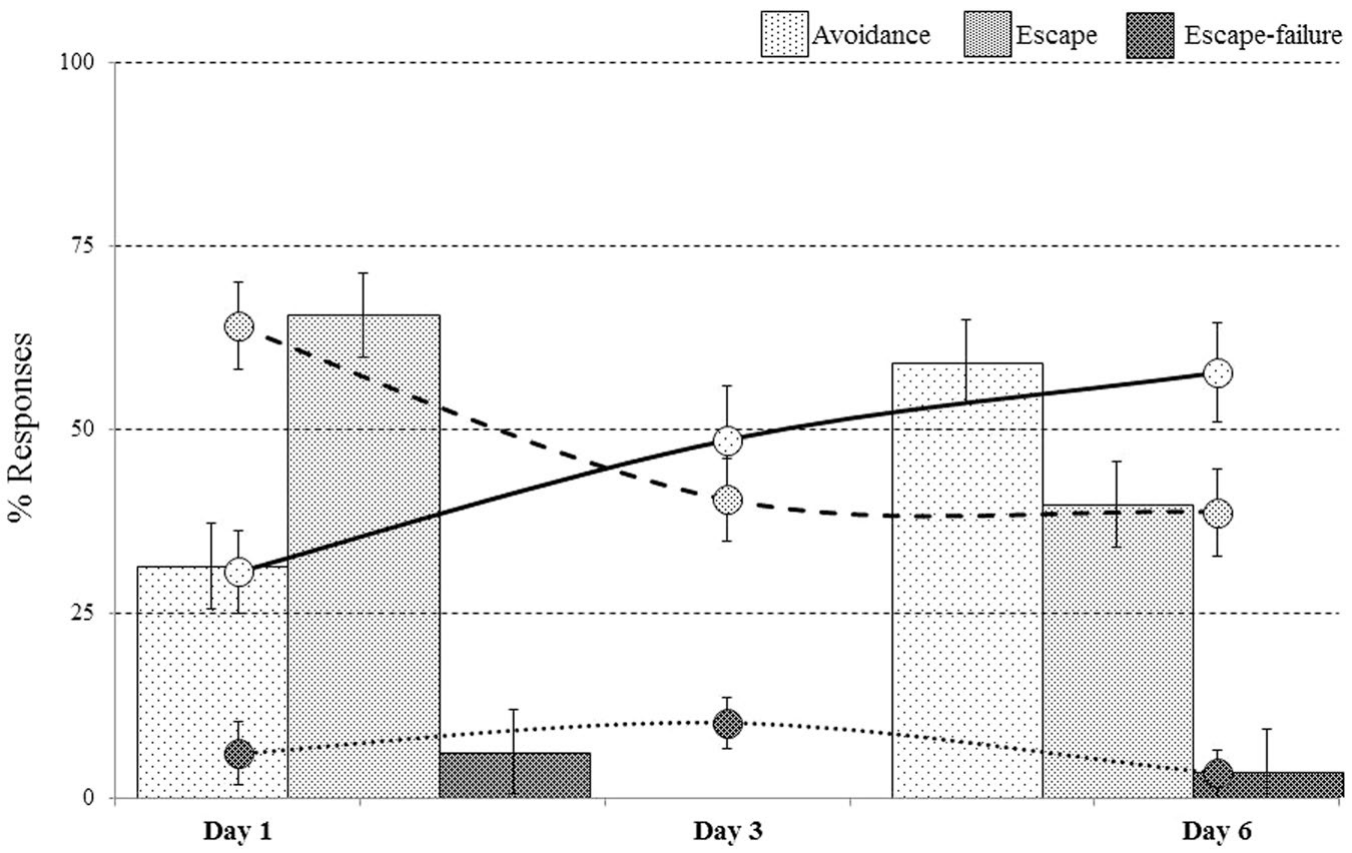
Performances in the ‘TWSA’ task: over the 6 days of training, controllable animals exhibited an increase in avoidance responses and a reduction in their escape responses. This demonstrates the rats’ ability to control the appearance of the US (* > 0.05, ** > 0.001).

A repeated measures ANOVA was conducted in controllable animals (*n* = 18). Mauchly’s test indicated a violation of the sphericity assumption [χ^2^(2) = 9.35, *p* = 0.009], and degrees of freedom were therefore corrected using Greenhouse-Geisser estimates of sphericity (ε = 0.70). Over 6 days of training in the TWSA, significant effects were found between the averaged behavioural responses, [F_(1.39,23.57)_ = 25.80, *p* < 0.001, η_p_^2^ = 0.60]. Bonfferoni corrected post-hoc comparisons revealed that in the 6^th^ day, controllable animals performed on average significantly more avoidances (57.78 ± 6.76%), less escapes (38.88 ± 5.93%) and fewer escape-failures (3.33 ± 3.1%) as compared with the average avoidances (30.72 ± 5.65%), escapes (64.07 ± 5.92%) and escape-failures (6.83 ± 4.32%) performed on the 1^st^ day [p < 0.05; Fig. 1 (bars)].

An additional repeated measures ANOVA was conducted for the learning curves during the training. Violation of the sphericity assumption was found in Mauchly’s test for avoidance responses [χ^2^(14) = 28.41, p = 0.014], and degrees of freedom were therefore corrected with Greenhouse-Geisser estimates of sphericity (ε = 0.58). A main effect was found for avoidance responses [F_(2.91,40.75)_ = 6.55, *p* < 0.001, η_p_^2^ = 0.32]. Sphericity was assumed for escape responses and a main effect was found [F_(5,70)_ = 10.10, p < 0.001, η_p_^2^ = 0.42]. Animals made too few escape-failure responses and therefore no statistical comparisons could be conducted. Bonfferoni corrected post-hoc comparisons revealed that over the 6 days of training, animals increased their avoidance responses while reducing their escape responses [*p* < 0.05; Fig. 1 (lines)].

Uncontrollable rats were exposed to tones and foot-shocks similar to those of the controllable group but their behaviour had no effect on the outcome in the TWSA (described in the methods). Naïve animals were handled for 10 min outside the *vivarium* during this time and were then returned to their home-cages.

### Performances in the test following the TWSA task

**Figure 2:**
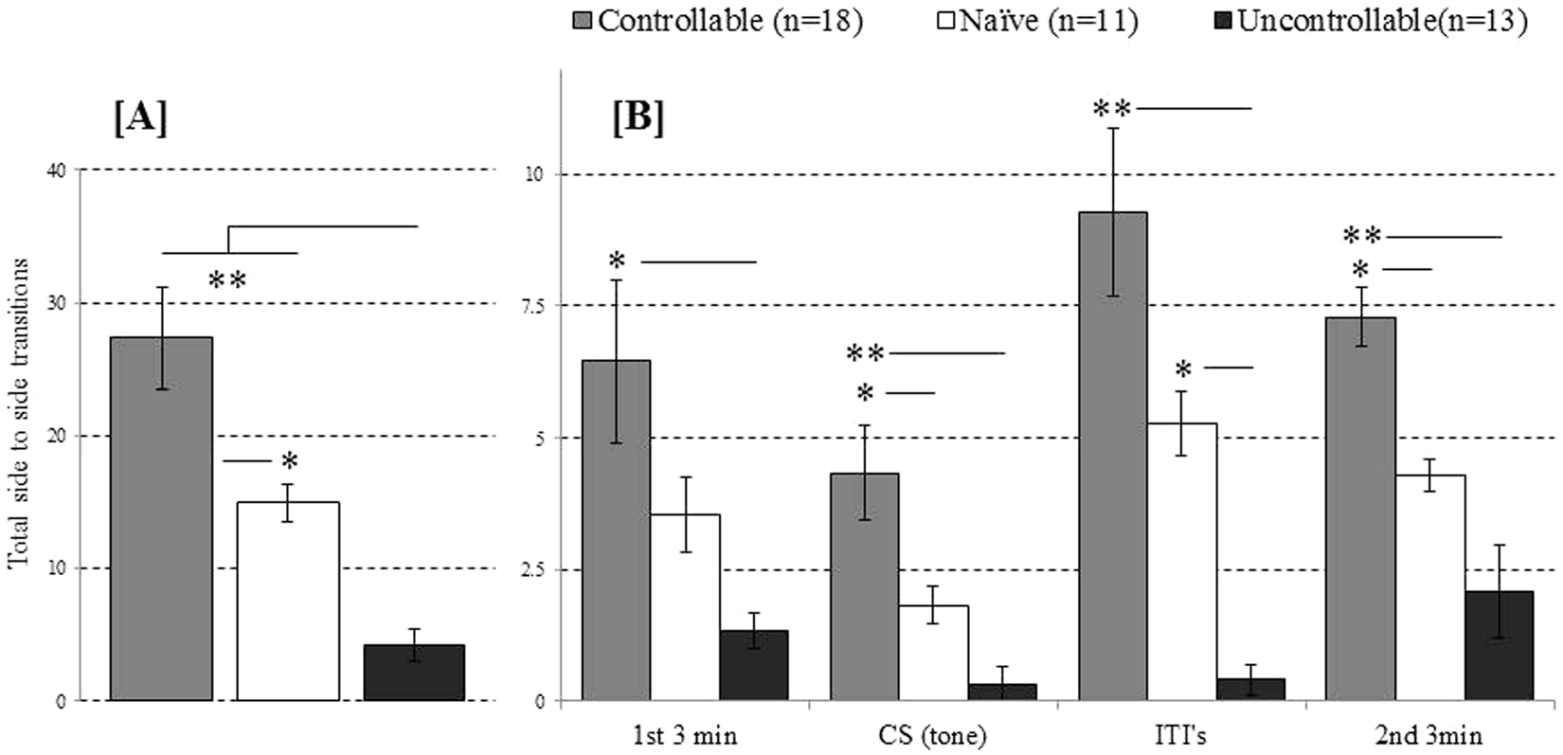
(**A**,**B**) Performances in the test after exposure to training in the ‘TWSA’ task: (**A**) controllable rats exhibited significantly more side to side transitions during the test, compared to naïve and uncontrollable rats. Likewise, uncontrollable rats exhibited significantly less transitions compared to naïve rats; (**B**) Significant differences in side to side transitions between controllable and uncontrollable rats were evident in each stage within the test (i.e. 1^st^ 3 min exploration, exposure to the CS, during ITI’s and during the 2^nd^ 3 min exploration; * > 0.05, ** > 0.001).

Two weeks following the exposure to the TWSA task, animals’ activity was assessed in the conditioned context (i.e. TWSA box) through a reminder of the CS (i.e., tone) but without the US (i.e., footshock). A one-way ANOVA for the effects of the TWSA exposure on activity in context revealed significant differences between the groups [F_(2,38)_ = 14.98, p < 0.001]. Figure 2-A depicts Bonfferoni corrected post-hoc comparisons, which revealed that both controllable (27.33 ± 3.87, *n* = 18) and naïve (14.91 ± 1.37, *n* = 12) animals performed significantly more side to side transitions during the 10 min test, compared to uncontrollable rats (4.16 ± 1.23; *n* = 13). Controllable rats also differed from naïve rats, [p < 0.05].

Activity during the different stages of the test was analysed by using a repeated measure ANOVA. Assumption of sphericity was violated as indicated by Mauchly’s test [χ^2^(5) = 24.52, p = 0.000], and Greenhouse-Geisser estimates were therefore used to correct degrees of freedom (ε = 0.71). Figure 2-B depicts the significant between group effects found for activity during stages within the test, [F_(4.37,83.04)_ = 15.58, p < 0.001, η_p_^2^ = 0.451]. Bonfferoni corrected post-hoc comparisons revealed that controllable rats performed significantly more side to side transitions during the first 3 min exploration (controllable: 6.44 ± 1.54; uncontrollable: 1.33 ± 0.33; naïve: 3.54 ± 0.72), exposure to the CS (controllable: 4.33 ± 0.90; uncontrollable: 0.33 ± 0.33; naïve: 1.82 ± 0.35), ITIs’ (controllable: 9.27 ± 1.60; uncontrollable: 0.41 ± 0.28; naïve: 5.27 ± 0.60), and the last (2^nd^) 3 min exploration (controllable: 7.27 ± 0.56; uncontrollable: 2.08 ± 0.86; naïve: 4.27 ± 0.30) [*p* < 0.05].

### Electrophysiological results

Within one week following the last behavioural assessment (i.e., PND 75), *in vivo* electrophysiology measurements were performed to explore whether levels of behavioural controllability would influence plasticity in the NAcc and BLA simultaneously^41^. In a previous study by our group, no effects were found for the dPAG HFS by itself^41^. Therefore, in the current study only the ability of dPAG priming + vSub HFS to differentially modulate BLA and NAcc plasticity was assessed.

Measurements of input-output (IO) curve responses were collected to determine the stimulation intensity needed for recording stable baseline responses. During IO-curves, stimulation range started at 0.2 mA and up to 1.8 mA with steps of 0.2 mA between one to the next. Although the NAcc and the BLA were recorded simultaneously, their statistical analysis is presented separately.

In the NAcc, an ANOVA with repeated measures was conducted on a within-subject factor (stimulation intensities) and a between-subject factor (groups). Mauchly’s test indicated that the assumption of sphericity had been violated [χ^2^(35) = 424.64, p = 0.000], and degrees of freedom were corrected using Greenhouse-Geisser estimates of sphericity (ε = 0.19). A main effect was found for stimulation intensities [F_(1.52,54.75)_ = 60.69, *p* < 0.001, η_p_^2^ = 0.628] but no effect was found for groups [F_(2,36)_ = 2.88, *n.s*., η_p_^2^ = 0.138] or for the interaction of stimulation intensities X groups [F_(3.04,54.75)_ = 2.59, *n.s*., η_p_^2^ = 0.039].

Likewise, in the BLA, a repeated measures ANOVA was used to assess a within-subject factor (stimulation intensities) and a between-subject factor (groups). Mauchly’s test indicated that the assumption of sphericity had been violated [χ^2^(35) = 311.19, p = 0.000], and degrees of freedom were corrected using Greenhouse-Geisser estimates of sphericity (ε = 0.32). A main effect was found for stimulation intensities [F_(2.53,91.23)_ = 73.77, p < 0.001, η_p_^2^ = 0.672] but not for groups [F_(2,36)_ = 2.83, *n.s*., η_p_^2^ = 0.136] or for the interaction stimulation intensities X groups [F_(5.07,91.23)_ = 1.43, *n.s*., η_p_^2^ = 0.073]. For averages and SEMs’, please see Table 1.

**Table 1:** Average measurements of input-output curve responses (mA/mV).

		BLA	NAcc
Naïve	vSub HFS (n = 6)	1.95 ± 0.28	1.99 ± 0.27
	0.5 mA dPAG priming + vSub HFS (n = 6)	2.52 ± 0.33	2.17 ± 0.42
Controllable	vSub HFS (n = 8)	1.95 ± 0.27	2.55 ± 0.23
	0.5 mA dPAG priming + vSub HFS (n = 8)	1.71 ± 0.42	1.98 ± 0.26
Uncontrollable	vSub HFS (n = 6)	3.10 ± 0.16	2.28 ± 0.94
	0.5 mA dPAG priming + vSub HFS (n = 6)	3.02 ± 0.93	2.31 ± 0.68

Averaged peak height amplitudes of the different experimental groups collected during input-output recordings for stimulation intensities calculation in BLA and NAcc.

### The effects of the ventral Subiculum (vSub) HFS on plasticity in the BLA and NAcc under controllable or uncontrollable conditions

#### NAcc

**Figure 3:**
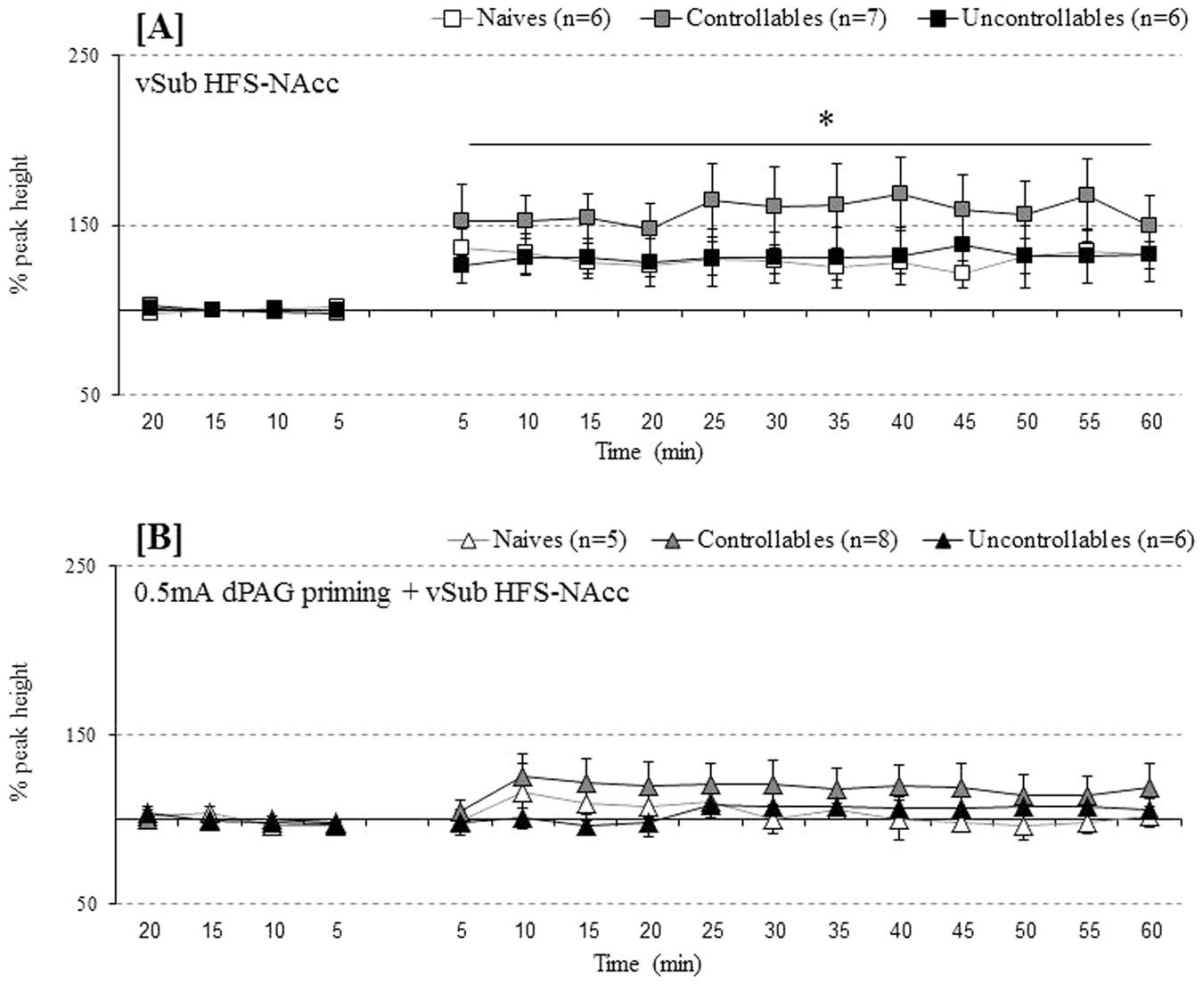
(**A**,**B**) Behavioural controllability effects on plasticity and metaplasticity in the NAcc: (**A**) Applying vSub HFS induced LTP in all examined groups (i.e. naïve, controllable and uncontrollable rats; * < 0.05 – compared to baseline); (**B**) Applying vSub HFS + 0.5 mA priming block the previously induced plasticity in the all groups (i.e. naïve, controllable and uncontrollable rats; *n.s*.).

A repeated measures ANOVA was conducted for a within-subject factor (time points during baseline before the application of HFS) and a between-subject factor (groups). Mauchly’s test indicated that the assumption of sphericity had been violated [χ^2^(5) = 27.84, p = 0.000], and degrees of freedom were therefore corrected using Greenhouse-Geisser estimates of sphericity (ε = 0.59). No effects were found for time points [F_(1.76,29.99)_ = 0.37, *n.s*., η_p_^2^ = 0.021], for groups [F_(2,17)_ = 1.19, *n.s*., η_p_^2^ = 0.123] or for the interaction of time points X groups [F_(3.53,29.99)_ = 29.67, *n.s*., η_p_^2^ = 0.172]. Similarly, an additional repeated measure ANOVA was conducted for a within-subject factor (time points) and a between-subject factor (groups) after applying HFS stimulation. Sphericity was assumed. A main effect was found for time points [F_(15,240)_ = 13.19, *p* < 0.001, η_p_^2^ = 0.452] but not for groups [F_(2,16)_ = 0.692, *n.s*., η_p_^2^ = 0.080] or for the interaction between time points X groups [F_(30,240)_ = 0.773, *n.s*., η_p_^2^ = 0.088]. As depicted in Fig. 3-B, all groups exhibited plasticity in the form of LTP following vSub HFS [*p* < 0.05].

#### BLA

**Figure 4:**
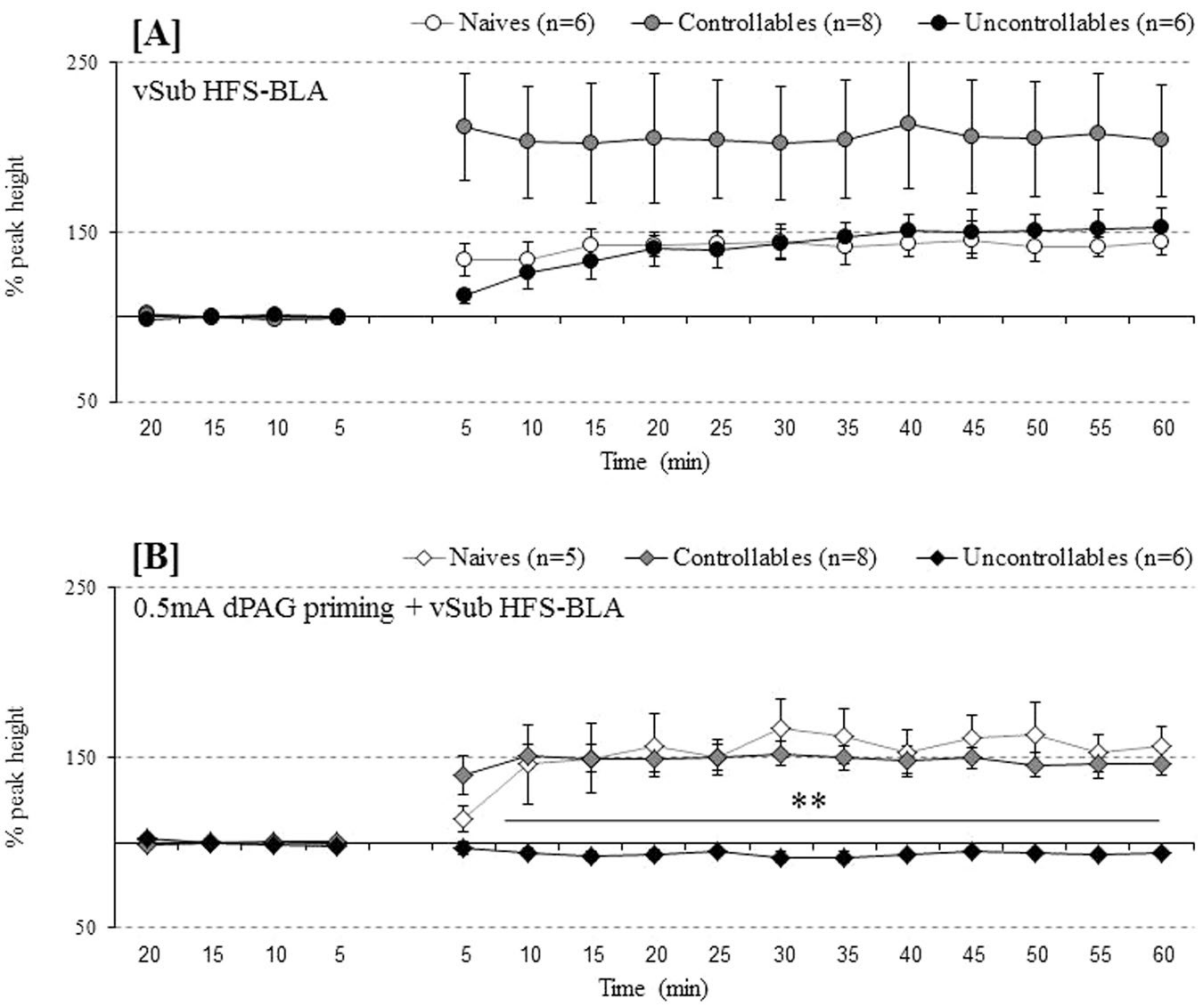
(**A**,**B**) Behavioural controllability effects on plasticity and metaplasticity in the BLA: (**A**) vSub HFS induce plasticity in the form of LTP in all examined groups (i.e. naïve, controllable and uncontrollable rats, p < 0.05). A trend for higher % plasticity was found in controllable rats compared to the other groups (i.e. naïve and uncontrollable animals, p = 0.06); (**B**) vSub HFS + 0.5 mA priming block this plasticity only in uncontrollable rats. Controllable and naïve rats both exhibited plasticity in the form of LTP (** < 0.001).

A repeated measures ANOVA was conducted for a within-subject factor (time points during baseline before the application of HFS) and a between-subject factor (groups). Mauchly’s test indicated that the assumption of sphericity had been violated [χ^2^(5) = 15.31, p = 0.000], and degrees of freedom were therefore corrected using Greenhouse-Geisser estimates of sphericity (ε = 0.62). No effects were found for time points [F_(1.87,31.74)_ = 4.92, *n.s*., η_p_^2^ = 0.013], groups [F_(2,17)_ = 0.531, *n.s*., η_p_^2^ = 0.059] or for the interaction of time points X groups [F_(3.73,31.74)_ = 18.33, *n.s*., η_p_^2^ = 0.091]. After applying HFS stimulation, an additional repeated measures ANOVA was conducted for a within-subject factor (time points) and a between-subject factor (groups). Again, Mauchly’s test indicated that the assumption of sphericity had been violated [χ^2^(119) = 636.37, p = 0.000], and degrees of freedom were therefore corrected using Greenhouse-Geisser estimates of sphericity (ε = 0.09). A main effect was found for time points [F_(1.31,22.22)_ = 16.78, p = 0.043, η_p_^2^ = 0.497] but not for groups [F_(2,17)_ = 2.45, *n.s*., η_p_^2^ = 0.224] or for the interaction of time points X groups [F_(2.61,22.22)_ = 25873.21, *n.s*., η_p_^2^ = 0.218]. As depicted in Fig. 4-A, all groups exhibited LTP following vSub HFS, and a LSD post-hoc comparison revealed a trend for increased plasticity in controllable rats (*n* = 8) compared to naïve (*n* = 6) and uncontrollable rats (*n* = 6), [*p* = 0.06].

### The effects of 0.5 mA dPAG priming on plasticity in the BLA and NAcc under controllable or uncontrollable conditions

#### NAcc

A repeated measures ANOVA was conducted for a within-subject factor (time points during baseline before the application of HFS) and a between-subject factor (groups). Sphericity assumed [χ^2^(5) = 10.00, p = 0.076]. No effects were found during baseline under the 0.5 mA dPAG priming condition for time points [F_(3,48)_ = 2.74, *n.s*., η_p_^2^ = 0.146], groups [F_(2,16)_ = 0.50, *n.s*., η_p_^2^ = 0.0.059] or for the interaction of time points X groups [F_(6,48)_ = 0.692, *n.s*., η_p_^2^ = 0.080]. After 0.5 mA dPAG priming + vSub HFS, an additional repeated measures ANOVA was conducted for a within-subject factor (time points) and a between-subject factor (groups). Mauchly’s test indicated that the assumption of sphericity had been violated [χ^2^(119) = 364.93, *p* = 0.000], and degrees of freedom were therefore corrected using Greenhouse-Geisser estimates of sphericity (ε = 0.15). No effects were found for time points [F_(2.29,36.76)_ = 1.83, *n.s*., η_p_^2^ = 0.103], groups [F_(2,16)_ = 1.169, *n.s*., η_p_^2^ = 0.336] or for the interaction of time points X groups [F_(4.59,36.76)_ = 0.965, *n.s*., η_p_^2^ = 0.108]. As depicted in Fig. 3-B, 0.5 mA dPAG priming before application of vSub HFS resulted in a failure to induce plasticity in all the experimental groups.

#### BLA

A repeated measures ANOVA was conducted for a within-subject factor (time points during baseline before the application of HFS) and a between-subject factor (groups). Mauchly’s test indicated that the assumption of sphericity had been violated [χ^2^(5) = 48.27, *p* = 0.000], and degrees of freedom were therefore corrected using Greenhouse-Geisser estimates of sphericity (ε = 0.39). No effects were found for time points [F_(1.19,19.06)_ = 0.980, *n.s*., η_p_^2^ = 0.058], groups [F_(2,16)_ = 1.09, *n.s*., η_p_^2^ = 0.120] or for the interaction of time points X groups [F_(2.38,19.06)_ = 36.95, *n.s*., η_p_^2^ = 0.143]. After applying 0.5 mA dPAG priming + vSub HFS, a repeated measure ANOVA was conducted for a within-subject factor (time points) and a between-subject factor (groups). Mauchly’s test indicated that the assumption of sphericity had been violated [χ^2^(119) = 497.06, *p* = 0.000], and degrees of freedom were therefore corrected using Greenhouse-Geisser estimates of sphericity (ε = 0.17). Significant effects were found for time points [F_(2.57,41.17)_ = 21.91, p < 0.001, η_p_^2^ = 0.578], groups [F_(2,16)_ = 15.86, p < 0.001, η_p_^2^ = 0.665] and for the interaction of time points X groups [F_(5.15,41.17)_ = 8.56, p < 0.001, η_p_^2^ = 0.517]. Post-hoc comparisons with Bonfferoni corrections revealed that uncontrollable rats (*n* = 6) failed to exhibit any plasticity compared to naïve (*n* = 6) and controllable (*n* = 8) rats that exhibited plasticity in the form of LTP [*p* < 0.05; Fig. 4-B].

### The interactive effects of group X stimulation protocols

Plasticity Indices (i.e., the ratio between the last 10 min baseline to the last 10 min following theta stimulation) were calculated for all groups. A two-way ANOVA for testing the interaction between groups (i.e., controllable, uncontrollable) and stimulation protocol (i.e., HFS, Priming + HFS) was insignificant, but two major main effects emerged.

#### Between groups comparisons

**Figure 5:**
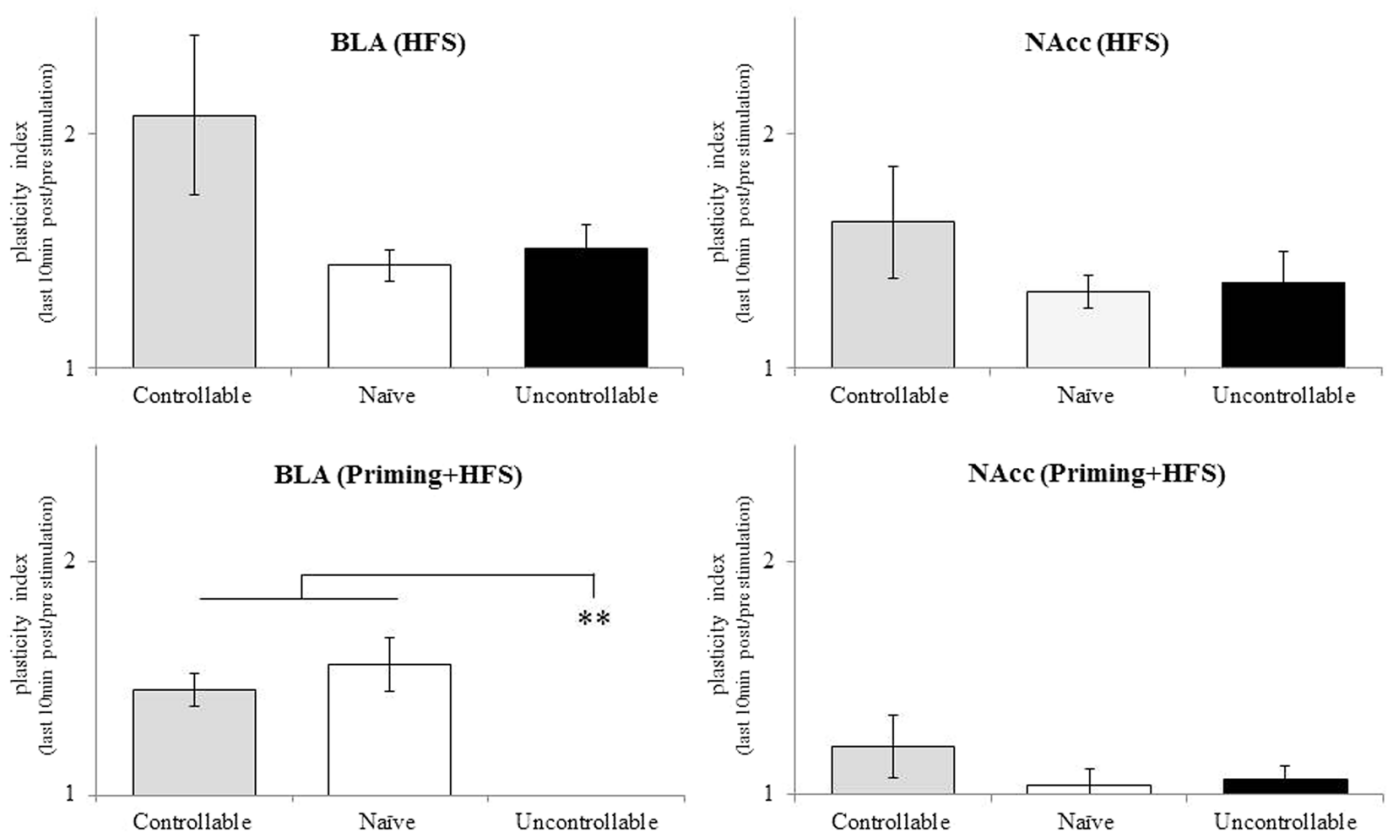
The interactive effects of group X stimulation protocols: between groups effects: under HFS stimulation a border-line significance for higher BLA plasticity index in controllable rats was evident compared to naïve rats (p = 0.08). Under 0.5 mA priming + HFS, both controllable and naïve rats exhibited higher BLA plasticity indices compared to uncontrollable rats (** < 0.001).

Figure 5 depicts the results of a one way ANOVA for testing the effects of group allocation on BLA and NAcc plasticity indices, which was significant for the BLA [F_(2,38)_ = 4.29, *p* < 0.05, η_p_^2^ = 0.211] but not for the NAcc [F_(2,38)_ = 1.59, *n.s*., η_p_^2^ = 0.090]. Further post-hoc examinations of this effect revealed that under HFS stimulation, borderline significance for higher BLA plasticity index in controllable rats was evident as compared to naïve rats [*p* > 0.05]. No differences were observed between the groups in NAcc plasticity index under HFS stimulation. Under 0.5 mA priming + HFS, both controllable and naïve rats exhibited significantly higher BLA plasticity indices compared to the uncontrollable rats [*p* < 0.05]. No differences were observed between the groups in NAcc.

#### Within group comparisons

**Figure 6:**
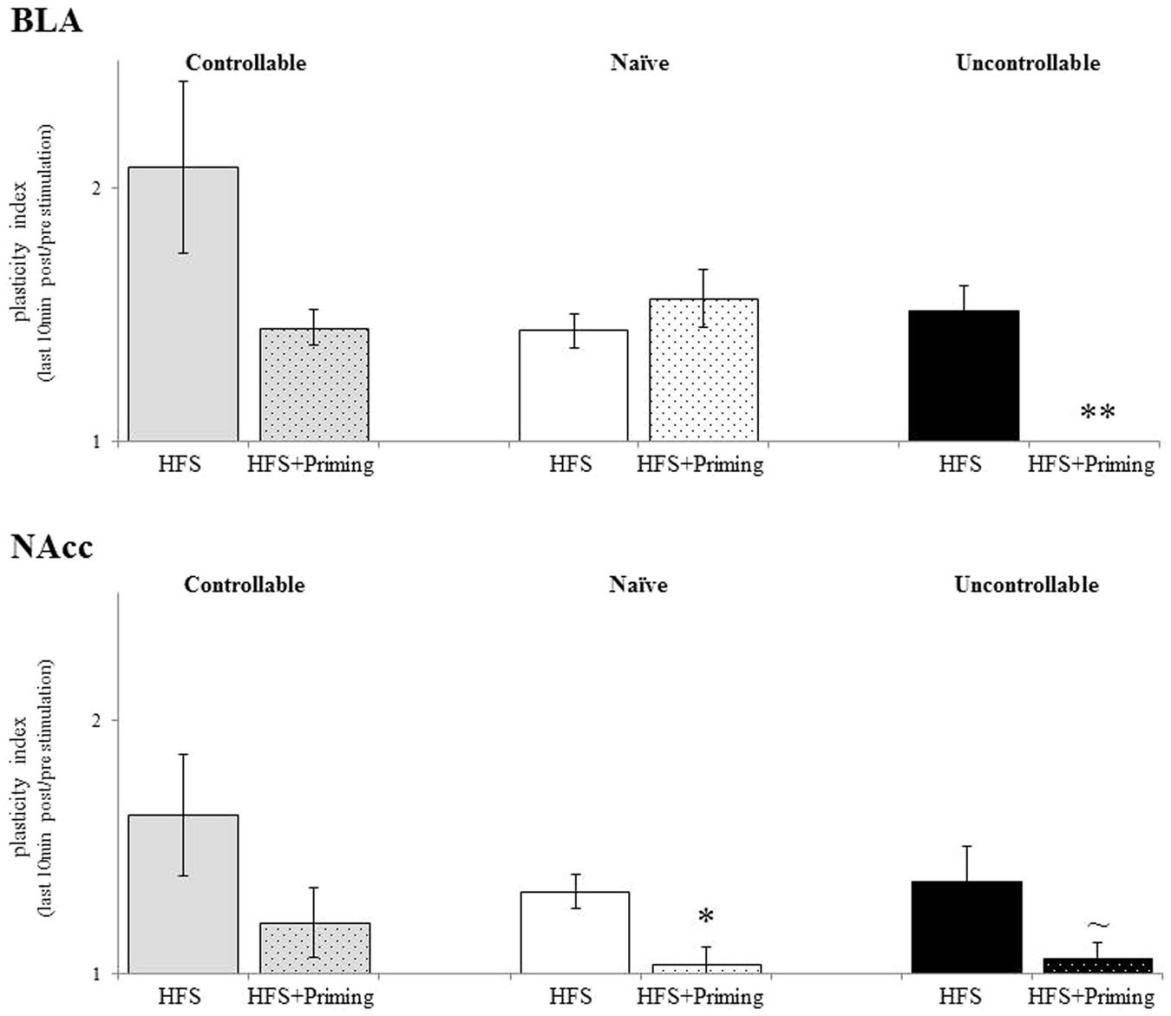
The interactive effects of group X stimulation protocols: within groups effects: a significant reduction in BLA plasticity index of uncontrollable rats following priming + HFS stimulation, compared to HFS stimulation was found (**p < 0.001). A trend was found for the NAcc plasticity index in this group [p = 0.07]. A significant reduction in NAcc plasticity index following priming + HFS stimulation, compared to HFS stimulation was found also in naïve animals [p < 0.05]. No changes in plasticity indices were observed in the controllable group, neither under HFS stimulation nor under priming + HFS stimulation.

In order to evaluate the impact of dPAG priming, a within group comparison for HFS with or without dPAG priming was conducted. A one way ANOVA was significant both for the BLA [F_(1,38)_ = 5.32, *p* < 0.05, η_p_^2^ = 0.142] and for the NAcc [F_(1,38)_ = 7.79, *p* < 0.05, η_p_^2^ = 0.196]. Additional post hoc comparisons revealed a significant reduction in the BLA plasticity index of uncontrollable rats following priming + HFS stimulation when compared to HFS stimulation only [*p* < 0.05]. A trend was also found for the NAcc plasticity index in this group [*p* = 0.07]. A significant reduction in the NAcc plasticity index following priming + HFS stimulation, as compared to HFS stimulation, was also found in naïve animals [*p* < 0.05]. In contrast, no changes in plasticity indices were observed in the controllable group, whether under HFS stimulation or under the priming + HFS stimulation, indicating that the impact of dPAG priming was reduced in this group as compared to the naïve and in particular the uncontrollable group (Fig. 6).

## Discussion

Previous research has shown that an individual’s level of controllability over a stressor is critical for the behavioural and neural processing of the experience^6, 7, 9, 10^. Behavioural controllability has been linked to activation of both *positive* and *negative*-affect related brain regions^21–23^ but traditionally their modulation has been studied separately. In the current study behavioural controllability or the lack there of (i.e., controllable vs. uncontrollable conditions) were used to assess simultaneous potential shifts in plasticity of *positive* and *negative*-affect related brain regions. Further, previous studies indicated that inescapable stress exposure induced ΔFosB in the PAG in mice^42^. Others reported that escapable shock in rats, in contrast to inescapable shock, increased activation in the dPAG (i.e. extracellular 5-HT)^43^. In accordance with the latter hypothesis, our group has recently found total activation (c-Fos-expressing cells) in the dPAG in stress-exposed animals^44^. Collectively, these results point to the PAG’s activation under exposure to different types of stressors. Thus, the ability of behavioural controllability to affect *metaplasticity* in these regions through dPAG priming was also examined.

Over prolonged training in the TWSA, controllable rats gradually learned to gain controllability over the appearance of the US. Beginning from the 2^nd^ day of training, animals increasingly performed more avoidance responses compared to escape responses and escape-failures. These results are in line with previous studies regarding gaining controllability over the course of prolonged exposure to the TWSA task^12, 14, 20^. Prolonged TWSA exposure induced different behavioural states as was evident in the test performed two weeks following the initial exposure phase. During this test, behavioural differences were conspicuous before, throughout and after an exposure to a reminder of the CS (within the conditioned context). A significant reduction in the number of ‘side to side’ transitions was found in the uncontrollable group, compared to the controllable and naïve animals. These differences were evident in each stage within the test. The current results along with previous studies^12, 20^ indicate that even after the cessation of exposure to the CS-reminder during the test (i.e., the 2^nd^ and last 3 min free exploration), uncontrollable animals continued to exhibit higher levels of immobility, indicating that uncontrollable rats did not show extinction of fear.

Further, it has been reported elsewhere that control over a shock blocked behavioural effects of later social defeat^15^. Others reported that fear-potentiation in the plus-maze was dependent on stressor controllability and contextual conditioning^42^. Taken together, gaining controllability over a stressor is a developing process that entails differences in behaviour through the course of learning. We previously have addressed the gradual development of emotional controllability following mastering controllability^12, 20^. In particular, it was demonstrated that while both controllable and uncontrollable groups have demonstrated similar levels of fear when brought back to the context of exposure after one day of training, this fear response was maintained only in the uncontrollable group following 6 days of training^12^. These results demonstrated the profound temporal differences in behaviour. Yet, the time passing from training to testing might in fact serve as a confounding factor. It was already shown previously that over the course of time, even a single stress exposure might augment long term responses (for example: ref. 45). Hence, this might also contribute to the results presented here. To what extent does such ‘incubation’ effect contribute under the current setting should be further examined in future studies. Nevertheless, the main aim of the current study was to assess dPAG ability to modulate a previously induced plasticity in the BLA and NAcc in well-developed controllability or uncontrollability. Thus, our main focus was on testing animals after these emotional states were fully established. This was verified by the marked behavioural differences observed between the two groups in the test.

Considering the accepted view that the BLA is associated more with responses to negative experiences and with negative affect^26, 27^ and that the NAcc is more involved in positive affect and in appetitive reinforcement learning^24, 25, 46^, it could be expected that the prolonged uncontrollable stress exposure would be associated with reduced plasticity in the NAcc and increased plasticity in the BLA. In addition, it could be hypothesized that the controllable state would be associated with the mirror effect, i.e., reduced plasticity in the BLA and increased plasticity in the NAcc. However, the emerging picture of the above associations is more complex. There was a tendency for a higher level of plasticity in the controllable versus the uncontrollable group, but this was not significant and furthermore was present both in the BLA and the NAcc [Fig. 5].

We have previously reported that the dPAG HFS fails to independently induce plasticity. Yet, HFS to the vSub enabled *metaplasticity* effects of dPAG priming to be revealed^40, 41^. Using 0.5 mA dPAG priming in the current study induced *metaplasticity* that suppressed the level of LTP that could be induced. This effect was also seen both in the BLA and the NAcc [Fig. 6]. Of note, the most significant suppression of LTP following dPAG priming was found in the uncontrollable group, and was observed in the BLA as opposed to the the NAcc [Fig. 5]. These findings may hint that the simplistic view presented above is inaccurate; the assumption that prolonged controllable stress would be associated with increased plasticity in the NAcc while uncontrollable stress would be associated with increased plasticity in the BLA, does not hold. Instead, a more complex mode of operation is suggested, which requires separating the analysis to two scenarios: without or with dPAG priming.

Without dPAG priming there is a coordinated impact of a prolonged training regimen, with a tendency for a facilitation of LTP level in the BLA among controllable animals. It should be noted that this tendency was not statistically significant and should be explored further to carefully verify whether this tendency is due to a small but evident effect, whether LTP is not consistently facilitated following prolonged training in the TWSA, or rather occurs as a result of large variances in recordings. Regardless, in relation to the current discussion the results imply that under these conditions there is no clear bias towards plasticity in either the BLA or the NAcc, and that a significant level of LTP was evident in both brain regions under both prolonged controllable and uncontrollable conditions. It can thus be concluded that under stressful conditions that do not involve the activation of the dPAG, no clear imbalance of plasticity is induced whether stress is controllable or uncontrollable.

dPAG priming reveals a significant difference between animals who have been exposed to either prolonged controllable or uncontrollable stress. dPAG priming suppressed NAcc plasticity in all groups, but this suppression was not statistically significant for the controllable stress group. However, only in the uncontrollable stress group did dPAG priming significantly suppress LTP in the BLA, forming a clear dissociation between the controllable and uncontrollable exposure conditioning.

This may indicate that under dPAG priming, with regards to the balance between the BLA and the NAcc, plasticity is differentially modulated by controllable or uncontrollable stress; under uncontrollable conditions dPAG priming suppresses LTP both in the BLA and in the NAcc, while under controllable stress conditions there is a bias for plasticity in the BLA. This stems from the fact that dPAG priming suppresses LTP in the NAcc but not in the BLA. According to the simplistic ‘BLA-Negative affect/NAcc-positive affect’ view, such a bias of plasticity in the BLA relative to the NAcc could be expected in the uncontrollable group but not in the controllable group. Thus, in line with recent additional studies^32, 33^, the results presented in the current study support a more complex model in which both the BLA and the NAcc systems contribute to both positive and negative affect.

We have previously demonstrated that in naïve animals the effect of dPAG priming was dependent on the intensity of stimulation^41^. A relatively strong priming intensity (1.0 mA) resulted in suppression of LTP in both the BLA and the NAcc, and even the appearance of long-term depression of the response. Milder intensity priming (0.5 mA, as was used in the current study) resulted in suppression of LTP in the the NAcc but not in the BLA^41^. The current results reverberate well with our previous findings. The effects of dPAG priming in the controllable group are similar to those of the milder intensity priming in the previous study, while the effects of dPAG priming in the uncontrollable group resemble those of the stronger intensity priming (1.0 mA) in the previous study despite the use of the milder priming intensity (0.5 mA). Indeed, classically, the PAGs’ functions are mainly attributed to *negative*-affect related behaviours (e.g., defensive responses such as flight, fight, and freezing; submissive postures; tonic immobilization; and autonomic arousal; for a review see: ref. 47). It is thus plausible to assume that prolonged uncontrollable stress exposure results in hypersensitivity to dPAG priming, which leads to priming effects with milder priming intensity that are similar to those observed with stronger intensity in naïve and controllable animals. The result is modulation of plasticity in the BLA and NAcc, which reflects more severe experiences even under mild conditions. This could underlie in part the greater difficulty of uncontrollable animals to cope with stressful challenges^20^.

The current exploratory study adds to the growing body of research on the effects of prolonged controllable exposure, which is associated with resilience to stress, and to the effects of prolonged uncontrollable exposure, which is associated with impaired coping abilities and depressive symptoms^20^. The study’s results indicate that prolonged controllable exposure is associated with a tendency for greater plasticity in both the BLA and NAcc. In contrast, prolonged uncontrollable exposure was found to lead to hypersensitivity of the NAcc and mainly the BLA to priming of the dPAG. These results suggest that both the BLA and the NAcc systems are involved in attempts to cope with stressful challenges, and that a differentiated function of their activation is associated with the end outcome. Metaplasticity in the dPAG-BLA-NAcc circuit induced by prolonged exposure to uncontrollable stress may underlie the related impaired ability to cope with emotional challenges^20^, and may contribute to psychopathologies associated with prolonged exposure to stress.

## Methods

### Ethical considerations

All experimental procedures were performed according to the NIH guidelines and were approved by the University of Haifa’s IRB committee.

### Housing

Rats were group housed in plastic cages (35 × 60 × 18 cm) on a bedding of sawdust. The vivarium maintains an automatic 12-hour light-dark cycle (on at 7:00 am). Water and food (Teklad Global Diet 20185, Harlan Teklad Ltd., WI, USA) *ad libitum*. All experimental procedures and assessments were performed during the light phase of the day.

### Subjects

Forty-three Sprauge-Dawley male laboratory rats (Harlan Laboratories Jerusalem) at PND 50 were used. Behavioral manipulations began at PND 55 and ended at PND 75. Forty rats underwent the electrophysiological assessments starting at PND 75 to PND 81. Three animals were excluded from the final electrophysiological analysis due to methodological issues.

### Experimental groups

Levels of controllability were obtained by using the TWSA task. Rats were randomly assigned to one of the following:

1. Controllable group: Rats underwent 6 days of *controllable* training in the TWSA task. Two weeks later, animals were tested in the training context while exposed to the CS tone. Within a week from the test day, all animals were electrophysiologically measured [*N*_total_ = 18; vSub HFS, *n* = 8; 0.5 mA dPAG priming + vSub HFS, *n* = 8].
2. Uncontrollable group: Rats underwent 6 days of *uncontrollable* exposure in the TWSA task, and were then tested as above [*N*_total_ = 13; vSub HFS, *n* = 6; 0.5 mA dPAG priming + vSub HFS, *n* = 6].
3. Naïve group: Rats were facilitated in the local vivarium throughout the whole experimental period until testing for activity in the training context and for electrophysiological assessments were conducted. Animals were handled and weighted once a week and during the other groups experiments [*N*_total_ = 12; vSub HFS, *n* = 6; 0.5 mA dPAG priming + vSub HFS, *n* = 6].

### Experimental design

Starting on the 6^th^ day and continuing for six consecutive days, animals underwent the TWSA task according to their groups. Two weeks later, animals were tested in the training context while exposed to the CS tone without US presentations. Within a week from the last behavioral assessment, animals were anesthetized and underwent the electrophysiological assessments by using either vSub HFS or 0.5 mA dPAG priming + vSub HFS protocols (detailed below). Immediately following the electrophysiological assessments, animals were decapitated and their brains were harvested for testing electrodes positioning by histology (i.e. Cresyl Violet staining).

## Behavioral manipulations and assessment

### Novel-setting exploration

Rats were placed in the TWSA apparatus (Model LE 916, Panlab S.L., Barcelona, Spain) while it was in an inoperative mode and were allowed to explore both compartments for a total of 10 min. Crossing over between compartments provided a basic index for the rat’s exploratory tendency.

### Two-way shuttle avoidance (TWSA) task

Immediately after the exploratory behaviour assessment a training session began.

#### Apparatus

the TWSA box, placed in a dimly lit, ventilated, sound attenuated room, is a rectangular chamber (51 × 25 × 24(h) cm − internal size) divided by an opaque partition with a small passage (8 × 8 cm) that connects two equal sized, side-by-side, cube shaped compartments. Both metal grid floors of the compartments are weight sensitive and electrifiable. Micro-switches transmit information about the location of the rat to a computer control and data collection program. This program controls both conditioned stimulus (CS) presentations (a tone produced by loud speakers located on the distal walls of the compartments) and unconditioned stimulus (US) – electric shock deliveries (to the animals’ feet through the compartment floor, by a Solid State Shocker/Distributor; Model LE 10026; Letica, Barcelona, Spain). Both CS and US deliveries were regulated by a Shutavoid software (Panlab S.L., Barcelona, Spain).

1. Controllable training: A single daily training session of 75 trials (on days 1 and 2), 50 trials (on days 3 and 4), and 25 trials (on days 5 and 6) was implanted. Each trial consisted of a 10 sec tone followed by a 10 sec electrical foot shock (0.7 mA) overlapping on the 9^th^ sec of the CS presentation (i.e., a trace conditioning protocol). The inter-trial interval (ITI) was 30 sec with variation of 25% from one ITI to another. Rats could perform one of the following responses: (1) **avoidance response**: shuttling to the adjacent chamber of the apparatus while the tone (CS) was on, thus avoiding the shock; (2) **escape response**: shuttling to the other compartment after the shock began (US), thus reducing exposure to the shock; (3) **no escape**: not shuttling to the adjacent chamber, thus receiving the full length of the shock (US).
2. Uncontrollable exposure: This group was subjected to the same trial schedule as the controllable group but the animals had no control over the stressor. A computerized program delivered the averaged protocols of tone/shock durations based on the performance of the controllable group in each day.

### Test

Two weeks after the end of the training, a test was performed: 3 min of exposure to the training chamber (1^st^ test exploration), followed by 10 trials consisting of 10 CS presentations (tone). Each CS was presented for 10 sec and was separated by an ITI of 30 sec (ITI) with variation of 25% from one to another; no foot shocks were presented during the test. Following the CSs’ presentations, an additional 3 min free exploration in the TWSA box was measured (2^nd^ test exploration). During the test, all groups were given the opportunity to make an instrumental (i.e., avoidance) response. The number of ‘side to side’ transitions during the 1^st^ exploration was referred to as the animals’ affective state, mainly for expressing the first responsiveness phenotype while re-encountering the previously exposed context. In addition, ‘side to side’ transitions were calculated for all stages within the test (i.e. 1^st^ test exploration, tone, ITI’s and 2^nd^ test exploration).

## Electrophysiological manipulations and assessments

### Surgical procedure

Rats were anesthetized (Urethane (0.5 mg/kg body weight), ip) and mounted in a stereotaxic apparatus (Stoelting Co. Illinois, USA). The scalp was incised and retracted, and head position was adjusted to place Bregma and Lambda in the same horizontal plane. Small burr holes (2 mm diameter) were drilled unilaterally in the skull for the placement of stimulating and recording electrodes. A 125 μm coated wire reference electrode was affixed to the skull in the area overlapping the nasal sinus. Placement of the stimulating electrodes was done according to the stereotaxic criteria and was based on our previous publications on these pathways^40, 41^. Stimulating electrodes were implanted in the dPAG and the vSub, and recording electrodes were implanted in the NAcc and BLA. During the course of experiments, body temperature was maintained at 36.5–37.4 °C with a feedback regulated temperature controller (FHC, Bowdoinham, ME, USA).

### Electrodes positioning

A stimulation electrode was positioned in the vSub (AP: −6.3 mm; ML: 5 mm; DV: [−6–−8 mm]). For priming the dPAG, an additional stimulating electrode was positioned in the dPAG (AP: −6.05; ML: 0.64; DV: −5.72). Recording electrodes were positioned in the BLA (AP: −3.2 mm; ML: 5 mm; DV: [−7–−7.5 mm]) and the NAcc (AP: 1.6 mm; ML: 0.9 mm; DV: [−5.5–−6.4 mm]).

### Electrodes characteristics

Bipolar concentric stimulating electrodes (125 μm; Kopf, Tujunga, CA) were used for stimulating the dPAG and the vSub. For recordings in the BLA and NAcc, we used stainless steel recording electrodes (tip diameter, 2 μm; 20 mm length; Plastic One Inc., model: E363/2/SPC ELEC.008-SS).

### Electrophysiological recording protocols

For testing vSub ability to induce plasticity in the BLA and NAcc, High Frequency Stimulation (HFS) train consisted of stimulating (the vSub) for 10 brief bursts (200 ms) of 100 Hz stimulation delivered at 1 Hz (a total of 200 pulses). A 20 min pre-HFS baseline was collected at stimulation intensity that elicited a field potential response that reached 35–40% of the maximum response collected during input-output recordings for both the BLA and the NAcc. Immediately following baseline, rats received 4 HFS trains separated by 5 min (i.e., ISI). Responses were collected (once every 20 sec) during the baseline session and for 60 min following the last stimulation session^41^. For testing dPAG priming on BLA and NAcc plasticity following vSub HFS, priming stimulation was composed of a single (0.5 mA) HFS train to the dPAG, delivered 10 sec before the application of HFS to the vSub.

### Calculating ratio peak height (PH)

In both the BLA and the NAcc, the principal measure of size of the averaged evoked field potentials was ‘peak to peak’ amplitude. Peak height amplitude was defined from the highest peak before a trough to the lowest peak.

## Calculating plasticity Index

Plasticity index refer to the ratio between the last 10 min baseline to the last 10 min following theta stimulation. Indices were calculated for the NAcc and the BLA separately in order to simply assess the plasticity during the electrophysiological recording with regard to the animal’s behavioural manifestations.

### Histology

At the completion of the electrophysiological assessment, rats were deeply anesthetized with a lethal dose of Urethane (0.5 mg/kg, into the heart), their brain was removed from their skulls. Brains were maintained in −80 °C. Serial 40 μm brain coronal sections were cut using a cryostat (−21 °C), mounted on gelatin-coated slides and stained with cresyl violet (5%, Sigma-Aldrich) to localize the electrodes sites by microscopic examination according to the atlas of Paxinos and Watson^48^.

### Statistical analyses

All statistics were conducted in SPSS 20.0. Initial tests were conducted using one way or repeated measures analysis of variance (ANOVA) All post hoc comparisons were made using the least significant difference multiple comparison tests. The results are expressed as means ± SEM, unless stated otherwise.
